# Gastric organoids—an in vitro model system for the study of gastric development and road to personalized medicine

**DOI:** 10.1038/s41418-020-00662-2

**Published:** 2020-11-22

**Authors:** Therese Seidlitz, Bon-Kyoung Koo, Daniel E. Stange

**Affiliations:** 1 Department of Visceral, Thoracic and Vascular Surgery, Medical Faculty and University Hospital Carl Gustav Carus, Technische Universität Dresden, Dresden, Germany; 2 Institute of Molecular Biotechnology of the Austrian Academy of Sciences, Vienna Biocenter (VBC), Vienna, Austria; 3 National Center for Tumor Diseases (NCT), Dresden, Germany: German Cancer Research Center (DKFZ), Heidelberg, Germany; Faculty of Medicine and University Hospital Carl Gustav Carus, Technische Universität Dresden, Dresden, Germany; Helmholtz-Zentrum Dresden - Rossendorf (HZDR), Dresden, Germany

**Keywords:** Cancer models, Experimental models of disease

## Abstract

Gastric cancer ranks as the fifth most common human malignancy and the third leading cause of cancer related deaths. Depending on tumor stage, endoscopic or surgical resection supported by perioperative chemotherapy is the only curative option for patients. Due to late clinical manifestation and missing reliable biomarkers, early detection is challenging and overall survival remains poor. Organoids are cell aggregates cultured in three-dimensions that grow with similar characteristics as their tissue-of-origin. Due to their self-renewal and proliferative capacity, organoids can be maintained long term in culture and expanded in many cases in an unlimited fashion. Patient-derived organoid (PDO) libraries function as living biobanks, allowing the in depth analysis of tissue specific function, development and disease. The recent successful establishment of gastric cancer PDOs opens up new perspectives for multiple translational clinical applications. Here, we review different adult stem cell derived gastric organoid model systems and focus on their establishment, phenotypic and genotypic characterizations as well as their use in predicting therapy response.

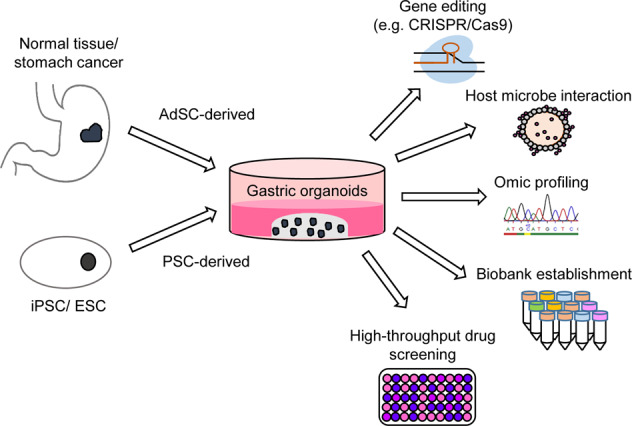

## Facts

- The stomach is an organ important for food processing, permanently in contact with nutrients and bacteria. To ensure a functional mucosa, a continuous self-renewal of the epithelium is required.
- Organoids are a three-dimensional cell culture system showing self-renewal, differentiation, and proliferation capabilities.
- Gastric organoids can be established from adult stem cells of primary tissue, embryonic stem cells or induced pluripotent stem cells.
- Gastric cancer ranks as the fifth most common world’s malignancy and the third leading cause of cancer related deaths.
- PDO biobanks of gastric cancer represent a useful tool in analyzing gastric cancer biology. PDOs allow individualized in vitro therapy and resistance testing.

## Open questions

- Is it feasible to generate and characterize cancer organoids between the patient’s cancer biopsy and the start of therapy in order to provide a therapy recommendation?
- Adult stem cell derived organoid cultures are purely epithelial. To what extent does this situation recapitulate the biological behavior in vivo?
- Is intra-tumoral heterogeneity present to various degrees in most cancers, a limiting factor for precision medicine, at least when organoids are derived from single biopsies with limited representation of the full genetic spectrum?

## The stomach—organ for food storage and digestion

### Anatomy, gland structure and function

**Fig. 1:**
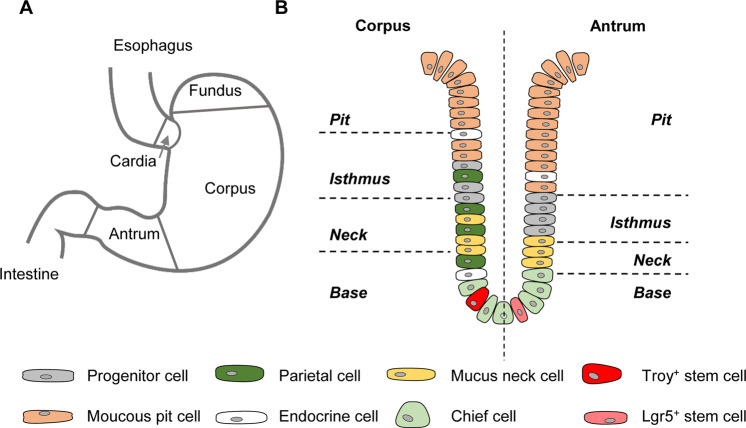
Morphology of the human stomach and gland organization. **A** The adult human stomach is divided into cardia, fundus, corpus (or body) and antrum ending in the pyloric sphincter. **B** Schematic representation of an adult corpus and antrum gland. The size and cell composition of the four regions pit, isthmus, neck and base differ between corpus and antrum.

The stomach is a muscular organ playing an important role in food storage and digestion. It consists of four main parts: cardia, fundus, corpus (body), and antrum (pylorus) (Fig. 1A) [1]. The corpus is the main part of the stomach secreting acid and digestive enzymes. The antrum plays an important role in hormone and mucus secretion. The murine stomach additionally possesses a squamous-epithelium lined forestomach important for storage and mechanical dissociation of food [2–5]. The stomach is in permanent contact to nutrients, toxins and bacteria that altogether generate a toxic environment [6]. To ensure an intact and functional mucosa, a continuous self-renewal of the epithelium is required.

The stomach mucosa is composed of an epithelial layer organized into glands. Four different regions are differentiated from bottom to top: base, neck, isthmus and pit. The corpus epithelium presents long glands with short pits containing mucus secreting pit and neck cells, acid secreting parietal cells, hormone secreting endocrine cells and digestive enzyme secreting chief cells (Fig. 1B) [7]. Antral glands are shorter, and have a relatively larger pit region. They also contain mucus secreting pit and neck cells as well as endocrine cells, but fewer basally located chief cells and no parietal cells [8]. The isthmus region contains proliferating cells and has long been considered the home of gastric epithelial stem cells [9]. As suggested by their names, mucus pit cells are found exclusively in the pit region and mucus neck cells in the neck region, whereas chief cells are located at the base region only. Hormone producing endocrine and acid secreting parietal cells scatter throughout the whole gland (Fig. 1B) [10–13].

### Gastric stem cells

The presence of stem cells allows the life-long self-renewal capacity of the stomach. In contrast to the small intestine, where *leucine-rich repeat-containing G-protein coupled receptor 5* (*Lgr5*) expressing cells at the crypt base constitute the undisputed stem cell population, the identity of the stomach stem cell is still under debate. The intestinal stem cell marker *Lgr5* was found to be also expressed at the bottom of the antral gland (Fig. 1B). Lineage tracing proved the stem cell properties of this cell type, as Lgr5^+^ cells self-renewed and were able to differentiate into all cell types of the antral epithelium [3, 14]. For the corpus gland, radiolabeling electron microscopy suggested a granule free undifferentiated cell within the isthmus [13, 15]. *Trefoil factor 2* (*Tff2*) mRNA transcript expressing cells, as opposed to TFF2 protein expressing neck cells, were shown to constitute short-lived progenitor cells of mucus neck, chief and parietal cells[16],. *SRY (sex determining region Y)-box 2* (*Sox2*) expressing cells have also been proposed to constitute long-lived stem cells of the gastric epithelium [17]. Nevertheless, *Sox2* is widely expressed throughout the gastric unit. A further study promoted *basic helix-loop helix family member a15* (*Bhlha15* or *Mist1*) expressing isthmus cells as stem cells of the stomach epithelium [18]. Of note, *Mist1* is also expressed by chief cells at the bottom of the glands. Cells expressing *the tumor necrosis factor receptor superfamily member 19* (*Tnfrsf19* or *Troy*) gene located at the gland base were shown by lineage tracing to self-renew and generate all cell types of the gastric gland, albeit inefficiently [19, 20]. Surprisingly, Troy^+^ cells are fully differentiated chief cells. Due to their quiescent, slow proliferating nature, which can be activated by tissue damage, Troy^+^ cells were proposed to serve as reserve stem cells [19]. This phenomenon opposes classical textbook knowledge, which describes an uni-directional differentiation flow of cells coming from stem cells toward differentiated cells, but might be a back-up mechanism active in several tissues [21]. It therefore appears that the “stem cell state” at least in certain stem cell populations can be influenced by the local microenvironment, which is subject to change in case of disturbance of homeostasis. A more general definition for a stem cell might therefore be: “A stem cell has the ability to replace lost tissue through cell division” [22].

In order to clarify the role and potential interplay of the two stem cell populations in the isthmus and base, Han et al. applied a detailed lineage tracing approach [23]. The isthmus stem cells, residing in the narrow zone between the pit and neck region, are multipotent and maintain the pit-isthmus-neck region by stochastic self-renewal, while the Troy^+^ stem cell population maintains chief cell regeneration at the gland bottom. This unifying concept was confirmed by a recent publication by Burclaff et al [24].

Similar to other tissues, gastric stem cells are discussed as the origin of gastric cancer. As they are long-lived, they are prone to accumulate mutations over time, eventually leading to aberrant signaling and finally full-blown gastric cancer. For the intestine, the Lgr5^+^ stem cell population has already been proven to constitute an important origin of intestinal cancer [25, 26].

## Gastric cancer

Gastric cancer ranks as the fifth most common world’s malignancy after cancers of the lung, breast, colorectum and prostate. The incidence rate decreased since the mid-20th century [27]. However, it remains the third leading cause of cancer related deaths worldwide [28, 29]. Adenocarcinoma of the esophagogastric junction (AEG) represents a cancer with clear histopathological and molecular overlap to gastric cancer. Of note, this entity shows a rising incidence rate over the last decades [30, 31]. Due to late clinical signs, diagnosis is delayed in three quarters of patients presenting with non-curable disease resulting in a poor overall prognosis [32]. Endoscopic or surgical resection is the only curative option, often supported by interdisciplinary approaches, i.e., perioperative chemotherapy. The most common conventional chemotherapeutic drugs used are fluoropyrimidines (i.e., 5-fluoruracil (5-FU), capecitabine, S-1), platinum compounds (i.e., cisplatin, oxaliplatin), docetaxel and epirubicin [33–35]. Besides the classical chemotherapy, genetic alterations represent molecular targets for potential targeted treatments. At the moment the only approved targeted therapies are trastuzumab, a monoclonal antibody against the human epidermal growth factor receptor 2 (HER2)/neu signaling, and the anti-vascular endothelial growth factor receptor (VEGFR) antibody ramucirumab [36, 37]. Other targeted therapies, like the anti-epidermal growth factor receptor (EGFR) antibodies cetuximab and panitumumab or the anti-VEGFR antibody bevacizumab, have so far failed to improve the survival rates. However, the latter studies were performed in mostly unselected patient cohorts due to missing biomarkers.

While the World Health Organization (WHO) classified gastric cancer into four main subtypes, the widely used Lauren classification divides gastric cancer into the intestinal, diffuse and intermediate subtype [38]. The intestinal subtype shows a glandular morphology of tumor cells and often metastasizes to the liver and lung. In contrast, the diffuse subtype is characterized by a non-coherent diffuse growth of cancer cells, characteristic signet-ring cells and a frequent metastatic spread to the peritoneum. In addition, the Cancer Genome Atlas (TCGA) study described a robust classification system into four subtypes based on molecular alterations [39]. The first group of gastric tumors is associated with an Epstein Barr virus (EBV) infection and is therefore termed “EBV subtype”. Tumors often show *phosphatidylinositol-4,5-bisphosphate 3-kinase catalytic subunit alpha* (*PIK3CA*) and *AT-rich interactive domain-containing protein 1* *A* (*ARID1A*) mutations, *cyclin-dependent kinase inhibitor 2* *A* (*CDKN2A*) silencing and a widespread hypermethylation of promotor regions. The second group of gastric tumors shows microsatellite instability (MSI) through hypermutation, *MutL homolog 1 (MLH1)* silencing and mutations of the DNA damage repair system. Accordingly, this group is termed “MSI subtype”. The third genetically defined gastric cancer cohort is named “chromosomal instability” (CIN) subtype. Cancer cells characteristically show an intestinal glandular histology with frequent *tumor protein 53* (*TP53*) mutations, receptor tyrosine kinase (RTK)-RAS pathway activation by receptor amplifications and a high amount of somatic copy number alterations (SCNA). The fourth molecular subtype shows a diffuse morphology of cancer cells due to the frequent loss of the *cell adhesion molecule cadherin 1 (CDH1)*. In addition frequent alterations are found in *Ras homolog family member A* (*RHOA*) and *ARID1A*. Due to a relatively low amount of SCNA, it is named “genomically stable” (GS) subtype.

## Organoids—a self-organizing and self-renewing three-dimensional cell culture model

Organoids are a recently developed three-dimensional (3D) cell cultivation system from adult stem cells (AdSCs) of primary tissue or embryonic stem cells (ESCs)/induced pluripotent stem cells (iPSCs) (together PSCs) [40, 41]. Cells are embedded in a laminin-rich extracellular matrix mimicking a native extracellular microenvironment. Some AdSC-derived organoids maintain or establish an intact stem cell niche, while others niche factors have to be supplemented in the medium. Stem cells in organoid cultures typically show a self-renewal and often an unlimited proliferative capacity. Depending on the amount of supplemented growth factors via the medium, cells within AdSC-derived organoids can differentiate into all or at least several differentiated cell types in the epithelium of the tissue they are derived from. PSC-derived organoids contain cells from different germ layers. Organoids typically exhibit physiological functions of the derived organs [41–45]. Therefore they constitute in many cases a near physiological cell culture model.

In 2009 the laboratory of Hans Clevers was the first to describe the AdSC-derived organoid system. In a seminal report, Sato et al. described a method to culture mouse intestinal organoids based on the knowledge of the specific growth factors required by intestinal stem cells [46]. The small intestinal epithelium is divided into crypts and villi as its main units [47]. The identification of the Lgr5^+^ stem cell located at the crypt base and their transcriptional profile formed the scientific basis of small intestinal organoid establishment [48, 49]. Mouse Lgr5^+^ stem cells were embedded in an extracellular matrix and overlaid with a medium containing the following niche factors: a WNT signaling agonist (Rspondin), epidermal growth factor (EGF) and Noggin. In general, WNT signaling is an essential requirement for crypt proliferation as well as for the maintenance of the intestinal stem cell pool [50–52]. WNT ligands are secreted by Paneth cells and stromal cells surrounding the intestinal crypt [53–55]. Within the intestinal murine organoid culture, which is a stroma-free cell culture system, Paneth cells constitute the essential WNT source for stem cells [56]. Rspondin is a potent WNT agonist enhancing WNT signaling via the LGR5/ring finger protein 43 (RNF43) axis [57]. Rspondin inhibits the action of RNF43/zinc and ring finger 3 (ZNRF3) by forming a complex with Rspondin and LGR4/5. This allows cells to express enough level of the WNT receptor Frizzled on the plasma membrane, which ensures constitutive WNT stimulation [58, 59]. Another important factor is EGF, which is important for epithelial proliferation and survival of undifferentiated cells [60–62]. EGF is secreted by Paneth cells [56], but still needs to be added to the intestinal organoid medium to allow long term growth. Bone morphogenetic proteins (BMPs) belong to the transforming growth factor-beta (TGF-β) family. TGF-β/BMP signaling inhibits epithelial proliferation and promotes differentiation in the intestinal crypt [63–65]. To prevent stem cell differentiation, Noggin as a BMP antagonist needs to be added to the growth medium. Within this culture setup, seeded single Lgr5^+^ cells are able to develop into mature organoids [46]. The intestinal organoid culture was later adapted to other mouse organs i.e., liver, lung, stomach, pancreas, prostate, endometrium, bladder, and salivary gland [14, 19, 66–77]. Organoids can also be grown from human tissue by adopting the growth requirements of murine culture protocols, allowing the establishment of large organoid collections [75, 77–84].

All those cultures have in common that they are grown in an extracellular matrix, which allows an outgrowth in 3D, and that they are cultured with a specific set of supplemented growth factors mimicking the niche microenvironment of each tissues and organs. Depending on the culture medium, differentiation of stem cells into organ-specific lineages is in part hampered by the presence of stem cell factors. For example, mouse colon organoids need a high concentration of WNT for their maintenance, which at the same time blocks differentiation of the stem cells [78]. Many cultures therefore consist of mainly stem and progenitor cells with rather immature differentiated cells, while fully differentiated cell types can only be observed after removing stem cell growth factors in the medium, resulting in the loss of stem cell driven longevity. Nevertheless, these stepwise culture conditions (expansion media and differentiation media) allows researchers to culture both stem/progenitor cells and differentiated cells. Further improvements of the culture conditions might resolve this problem in the future. For example, an improved medium condition by adding insulin-like-growth-factor-1 (IGF-1) and fibroblast growth factor-2 (FGF2), instead of adding EGF to the growth medium, preserved cellular diversity in normal human intestinal organoids, leading to the presence of stem and progenitor as well as differentiated goblet cells and a few Paneth cells [85].

The main advantage of the organoid culture system over most other primary cell cultures is the possibility to maintain the genomic stability of cells for a long period of time while retaining the characteristics of the tissue-of-origin [86, 87]. Organoids can be split, frozen and thawed like the traditional 2D cultures. All these advantageous features make organoids a useful technology for many laboratory applications. Organoid cultures are compatible to molecular characterization by genomic, (single cell) transcriptomic and (large-scale) proteome analyses. It is also possible to apply genetic manipulations using lentiviruses, bacterial artificial chromosomes (BAC) or the clustered regularly interspaced short palindromic repeats (CRISPR/Cas9) system [80, 83, 88–96]. Bacterial or viral infections into the lumen of organoids play an important role for studying infectious diseases [97–99].

In conclusion, the current organoid technology enables patient-derived organoid collections, genetic manipulations, and various omics studies, opening up unprecedented new possibilities for individualized treatment testing and in depth analysis of the underlying pathobiology. In particular, with the help of organoid biobanks, high-throughput drug screenings can be performed to identify novel treatment options [100].

## Gastric organoids

### Adult stem cells and pluripotent stem cells allow the establishment of gastric organoids

Gastric organoids may be initiated from stomach tissue derived AdSCs as well as PSCs. The main difference between the systems is the presence of mesenchymal cells within the PSC-derived organoid culture. AdCSs can only generate the specific cells from the tissue-of-origin, whereas PSCs have by nature the ability to differentiate into any cell types. Therefore, PSC-derived organoids require a stepwise differentiation protocol that guides PSC differentiation into the target tissue identity, while AdSC-derived gastric organoids from the start only require a single growth factor-enriched medium. Consequently, the time to differentiate PSCs into organoids takes ~30–60 days, while AdSC-derived organoids are established within 7–14 days.

**Fig. 2:**
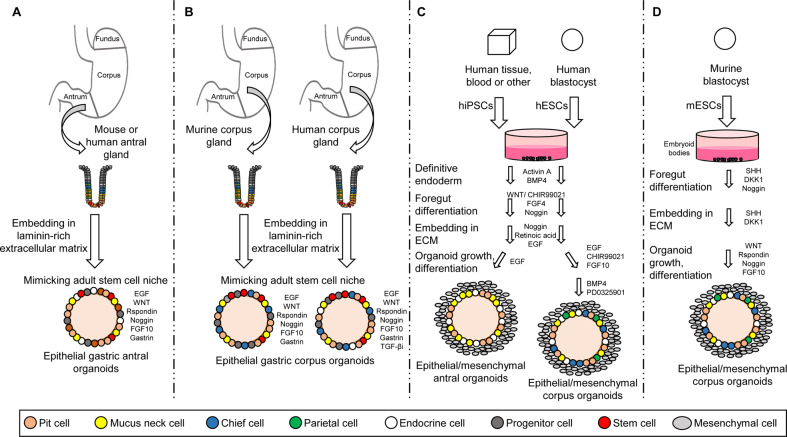
Generation of stomach organoids from adult stem cells (AdSCs) and pluripotent stem cells (PSCs). **A** Mouse or human antral organoids and (**B**) mouse or human corpus organoids are initiated by isolating stomach glands, embedding them in extracellular matrix and supporting them with growth media composed of EGF, WNT, Rspondin, Noggin, FGF10 and gastrin. The inhibition of the TGF-β signaling pathway increased the longevity of human corpus organoids. **C** Differentiation of PSC-derived human antral and corpus organoids. PSCs were obtained from blastocysts (embryonic stem cells (ESC)) or by reprogramming of differentiated cells (induced PSCs (iPSCs)). Cells were differentiated into endoderm by addition of activin A and BMP4. Posterior foregut formation was reached by supplementing with FGF4 and WNT or CHIR99021. Noggin was added for foregut differentiation. Embedding of these cells into an extracellular matrix led to the generation of 3D foregut spheroids. Antral differentiation was achieved by retinoic acid (RA) and EGF treatment. To direct foregut into corpus lineage the organoids were cultured with CHIR99021, EGF and FGF10. **D** Differentiation of PSC-derived mouse corpus organoids. PSCs were cultured as embryoid bodies and exposed to sonic hedgehog (SHH), the WNT antagonist dickkopf 1 (DKK1) as well as Noggin. Embedding of spheroids into extracellular matrix with addition of FGF10, Noggin, WNT and Rspondin led to mouse corpus gland formation.

The first murine adult stem cell derived stomach organoid culture was established from antrum glands containing Lgr5^+^ stem cells. The protocol was developed on the basis of the intestinal organoid culture system by the addition of fibroblast growth factor 10 (FGF10) and the hormone gastrin (Fig. 2A) [14]. In addition, markers of chief cells (pepsinogen C (PGC)) and mucus neck cells (MUC6) could be observed. Reduction of the WNT concentration resulted in the generation of the differentiated lineages of mucous pit and endocrine cells, while parietal cells were not observed [14]. The same conditions were later used for murine corpus organoids originating from Troy^+^ stem cells (Fig. 2B) [19]. These organoids expressed markers of chief cells and mucus neck cells. Upon the withdrawal of WNT, Noggin and FGF10 differentiated pit cells could be observed, but no endocrine or parietal cells.

Human antral organoids can be established using the mouse protocol (Fig. 2A) [101]. Human corpus organoids need the inhibition of the TGF-β signaling pathway by A83-01 (activin receptor-like kinase (ALK 5) inhibitor) for successful long term growth (Fig. 2B) [99].

The differentiation of PSCs into organoids allows the establishment of gastric organoids containing both epithelial and mesenchymal cells. McCracken et al. described the first differentiation protocol of human PSCs into gastric organoids (Fig. 2C) [102, 103]. Firstly, human PSCs were differentiated into endoderm by addition of activin A and BMP4. Activin A stimulates Nodal signaling, a highly conserved pathway important for foregut formation. Posterior foregut formation was achieved by addition of FGF4 and WNT or CHIR99021, a glycogen synthase kinase 3(GSK3)/β inhibitor stimulating the WNT pathway. To generate foregut, from which the stomach derives, Noggin was additionally applied and led to inhibition of BMP signaling. Embedding of these cells into an extracellular matrix led to the generation of 3D foregut spheroids. Antral differentiation was achieved by retinoic acid (RA) and EGF treatment. The complete differentiation required ~34 days resulting in antral organoids containing pit, mucus neck, and enteroendocrine cells [102, 104]. To direct foregut into corpus, the organoids were further supplemented with CHIR99021, EGF, and FGF10. To stimulate the production of parietal cells, the medium was supplemented subsequently with BMP4 and the MEK inhibitor PD0325901 (Fig. 2C) [103]. Differentiated corpus organoids contained pit, mucus neck, endocrine, chief and parietal cells [103, 104]. In a similar way, using a stepwise differentiation protocol, Noguchi et al. successfully generated organoids from murine PSCs (Fig. 2D) [105]. Here, the PSCs were cultured as embryoid bodies and exposed to sonic hedgehog (SHH), the WNT antagonist dickkopf 1 (DKK1) as well as Noggin. SHH activation as well as WNT signaling inhibition allowed tube-like structure formation and the generated spheroids resembled early stomach like structures. Embedding of spheroids into extracellular matrix with addition of FGF10, Noggin, WNT and Rspondin led to corpus gland formation after ~60 days. Similarly to the human PSC-derived corpus organoids, the murine PSC-derived organoids contained pit, mucus neck, endocrine, chief and parietal cells (Fig. 2D) [105].

In order to overcome the limitation of AdSC-derived organoids with its purely epithelial composition, a co-cultivation protocol of murine AdSC-derived organoids with mesenchymal cells was established [106]. The presence of mesenchymal niche cells resulted in the generation of all cells of the stomach epithelium including parietal cells (although only for a limited time span).

Overall, gastric normal organoids represent an excellent model system to answer questions of a wide range of topics, from basic science to translational clinical studies. Organoids can i.e., be used to recapitulate organ development [107]. They represent a useful tool to study *Helicobacter pylori* infection [108, 109]. Furthermore, organoids have been used successfully for disease modeling using CRISPR/Cas9 [110]. We will further focus on the establishment, characterization, and analysis of patient-derived gastric cancer organoid biobanks.

### Patient-derived gastric cancer organoids

Cancer organoids as avatars of a patient’s tumor hold a great promise. The individual cancer organoid can be used to predict therapeutic responses to certain drugs, while the establishment of large PDO biobanks in combination with drug screens might be useful to delineate novel therapeutic strategies in gastric cancer in general.

Recently, four independent groups reported the generation of gastric PDOs [81–84] (Table 1). The protocol to culture cancer organoids was based on the described protocol for normal gastric tissue organoids [99]. Tissue samples from histologically confirmed (metastatic) gastric or esophagogastric junction adenocarcinoma were obtained from surgical resection specimens as well as endoscopic, ultrasound- and computed tomography (CT)-guided biopsies or ascites punctures (Table 1) [81–84]. Seidlitz et al. generated a biobank composed of 20 different human gastric cancer organoids with an in depth molecular analysis of four lines [81]. Vlachogiannis et al. generated a PDO biobank including cancers of different gastrointestinal origin, incl. four gastric cancers [82]. Of note, the organoids were generated from patients within clinical trials, allowing a correlation of patient to organoid response. Nanki et al. generated a biobank of 37 molecularly characterized gastric cancer organoids [83]. The study convincingly established a direct link between mutation pattern and growth factor independency in the culture medium, highlighting the different niche dependencies of individual cancers. The largest biobank was generated by Yan et al., consisting of 46 molecularly characterized organoid lines [84]. Interestingly, this study also compared organoids derived from multiple biopsies of the same patient, thus allowing the analysis of subclones within the primary cancer.

**Table 1:** Overview of patient derived gastric cancer organoid library characteristics.

	Vlachogiannis et al.	Seidlitz et al.	Yan et al.	Nanki et al.
**Tissue source**				
	Ultrasound and CT-guided biopsy	Surgical resection	Surgical resection	Surgical resection
				Endoscopic biopsy
	Endoscopic biopsy			Ascites punctures
**Organoid library**				
Cancer	4	20	46	37
Normal	n/s	3	17	6
**Involvement of patients within clinical trials**				
	Yes	No	No	No
**Enzymatic tissue digestion**				
	EDTA (1 mM)	Dispase II (1 mg/ml)	Colagenase (0.6 mg/ml)	Liberase TH
	TrypLe (2x)	Collagenase XI (0.1 mg/ml)	Hyaluronidase (20 µg/ml)	TrypLe Express
**Media composition**				
WNT3A	100 ng/ml	50%	50%	25%
Rspondin	500 ng/ml	10%	10%	1 µg/ml
Noggin	100 ng/ml	10%	10%	100 ng/ml
B27	1x	1x	1x	1x
N2	1x	1x	n/s	–
Nicotinamide	4 mM	10 mM	n/s	–
N-acetyl-L-cysteine	n/s	1 mM	1 mM	1 mM
hFGF10	10 ng/ml	200 ng/ml	200 ng/ml	50 ng/ml
hFGF10-basic	10 ng/ml	n/s	n/s	n/s
mEGF	50 ng/ml	50 ng/ml	50 ng/ml	50 ng/ml
Gastrin	10 nM	1 nM	1 nM	10 nM
A83-01	0.5 µM	2 µM	2 µM	500 nM
Y-27632	10 µM	10 µM (only for organoid initiation)	10 µM	n/s
Prostaglandin E2	1 µM	n/s	n/s	n/s
SB202190	5 µM	n/s	n/s	n/s
**Cancer organoid selection to prevent normal organoid overgrowth**				
	No	No	Yes	Yes
Selection via			1. Microscopically organoid picking	1. + Nutlin3a (3 µM) - Y-27632
			2. Nutlin3a (10 µM)	2. - A83-01 + TGFβ (10 ng/ml)
				3. - EGF and FGF10

n/s not specified.

**Fig. 3:**
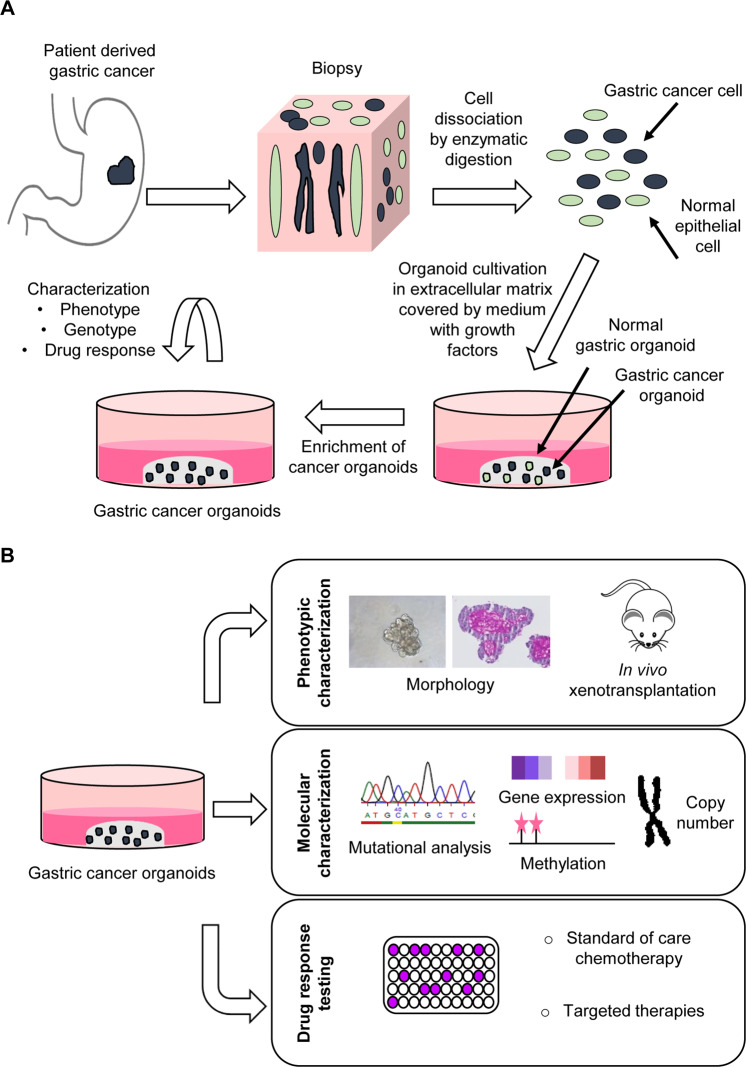
Patient-derived gastric cancer organoid generation and characterization. **A** Scheme of PDO culture generation from a cancer biopsy: enzymatic digestion, embedding in extracellular matrix, addition of growth medium and cancer organoid enrichment by media compound withdrawal and/or addition of mutation related inhibitors. **B** Overview of PDO characterization based on morphology, molecular pattern, and drug response testing.

The general setup for gastric cancer organoid establishment followed the same lines in all studies. After obtaining the tissue, the specimen is enzymatically digested, extracted cells are embedded in extracellular matrix and overlaid with medium (Fig. 3A). Different protocols for the enzymatic digestion have been described: incubation with dispase II and collagenase XI [81], EDTA and TrypLe [82], Liberase TH and TrypLe Express [83] or collagenase and hyaluronidase [84]. Growth media composition also varied slightly within the studies (Table 1). A general problem in cancer organoid generation is the “contamination” of the culture with normal cell-derived organoids from the specimen. Different strategies have been used to enrich for cancer organoids [111]. In an elegant way, Nanki et al. enriched cancer organoids by blocking the frequently altered signaling pathways i.e., TP53, RHO, TGF-β and RAS-phosphoinositide 3-kinase (PI3K), which non-mutated normal organoids do not tolerate [83]. This resulted in an increase of cancer organoid generation efficiency from ~55 to 75% [83]. Yan et al. enriched tumor organoids by microscopical selection and, in case of TP53 mutation, by nutlin3a treatment (Table 1) [84].

The further parts of this review focus on the characterization of patient derived gastric cancer organoids concerning phenotypic and molecular characterization as well as drug response testing (Fig. 3B).

#### Phenotypic characterization

Individual PDOs show different growth patterns characteristic to each line. Seidlitz et al. documented cystic organoids with or without a thickened (multilayered) wall, a non-coherent grape-like growth pattern and organoids with a compact cell cluster with no lumen [81]. The studies of Nanki and Yan grouped the divergent morphologies into three subtypes and correlated them to the Lauren classification: a solid-subtype derived mainly from diffuse gastric cancer showing amorphous solid configurations and a discohesive growth pattern, a glandular-subtype derived mainly from intestinal cancer showing glandular structures with a single lumen, and the mixed-subtype [38, 83, 84]. Cancer organoids also varied in proliferation rate, in line with different growth characteristics seen in patients [81]. Xenotransplantation of PDOs confirmed the tumorigenic potential of generated organoids and additionally the recapitulation of primary cancer morphology and histological subtypes [81, 83]. The link between phenotype and genotype was elegantly demonstrated by Nanki et al. by performing a knockout of *CDH1* using CRISPR/Cas9. The *CDH1* knockout led to a phenotypical change of organoids from normal cystic structures to solid structures with a vigorous migratory activity resembling PDOs with *CDH1* mutation [83]. This experiment nicely demonstrates the usefulness of the organoid system in combination with genetic engineering to gain insights into phenotypical and functional mechanisms of certain mutations.

#### Molecular characterization

The availability of high throughput sequencing modalities allows molecular profiling by genetic, transcriptomic, and epigenetic analyses outlining the (epi)genetic landscape of the individual tumor. Observed mutation patterns of gastric cancer PDOs recapitulate the described TCGA stomach cancer subtypes, and PDOs of all subtypes can be generated [39]. PDO cultures of the MSI, GS, CIN as well as EBV subtype could be established [81, 83, 84]. Furthermore, a 96% overlap in the mutational spectrum of organoid and parental tissue was found [82]. One potential pitfall when using PDOs as avatars of a patient’s tumor is its derivation from a biopsy or small piece of tissue from a resection specimen. Intra-tumoral heterogeneity might play an important role in therapy resistance development out of underrepresented but resistant subclones. The generation of organoid lines from different areas of resection specimens in colorectal cancer has unraveled such intra-tumoral heterogeneity [112]. It will be crucial to clarify the importance of intra-tumoral heterogeneity for therapy response testing in the future. As all subclones within one tumor often harbor a common mutational origin, the arising differences during cancer progression might not necessarily lead to a differential response among subclones. If intra-tumoral heterogeneity turns out to be crucial for therapy response prediction, taking several biopsies could be a solution, but might not be ethically feasible which could limit the potential clinical usefulness of PDOs. Yan et al. observed varying degrees of tumor heterogeneity in gastric cancer by comparing PDOs from primary tumor and lymph node metastasis [84]. A PDO library of different tumors (i.e., primary and several metastases) of an individual patient constitutes a novel research tool to study the consequence of intra-tumoral heterogeneity.

**Fig. 4:**
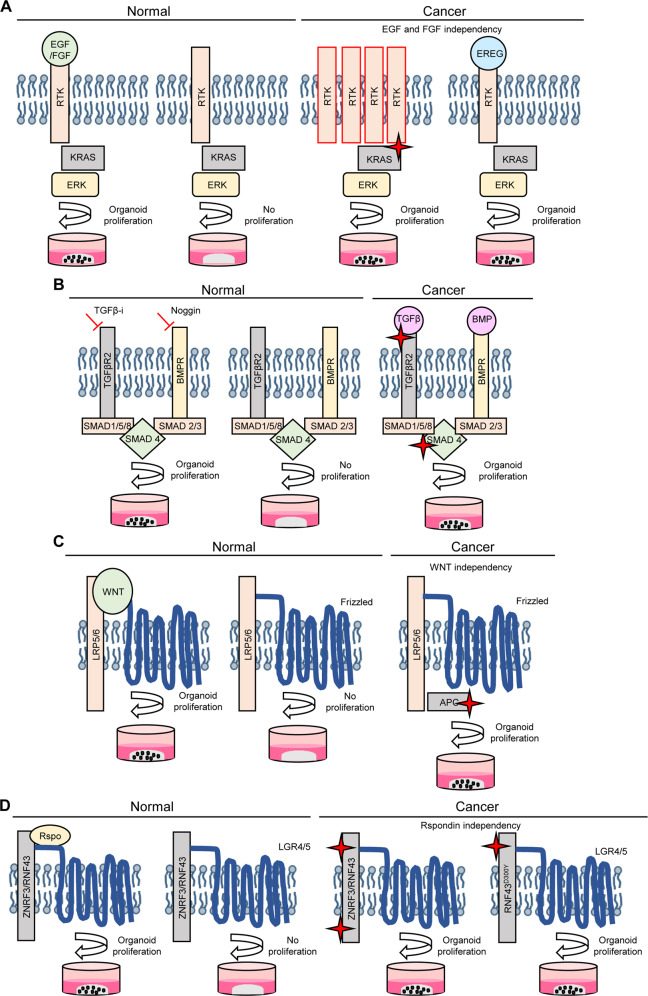
Altered pathways lead to growth factor independence of gastric cancer cells. **A** EGFR/FGFR signaling pathway in normal and gastric cancer organoids. Normal gastric cancer organoids are dependent on EGF/FGF10. *KRAS* mutation or receptor tyrosine kinase (RTK) amplification lead to an EGF/FGF10 independency. The upregulation of the EGFR ligand epiregulin (EREG) also mediates EGF/FGF independency. **B** TGF-β and BMP signaling in normal and cancer gastric organoids. In normal gastric organoid cultures, TGF-β and BMP signaling has to be inhibited to avoid differentiation and ensure proliferation. Cancer organoids with mutations in these pathways tolerate TGF-β and BMP signaling. **C** WNT signaling in normal and gastric cancer organoids. WNT receptor stimulation is important for organoid proliferation. Cancer organoids with *APC* alterations develop WNT independency. Porcupine plays an important role in WNT ligand production. Inhibition of this protein revealed dependencies on autocrine WNT loops. **D** Rspondin/ZNFR3/RNF43 signaling in normal and gastric cancer organoids. Rspondin is essential for normal gastric organoid growth. Alterations of *ZNFR3* and *RNF43* or the single *RNF43*^*D300Y*^ mutation results in Rspondin independence.

PDO survival under growth factor withdrawal of relevant media compounds can indicate an acquired independency of certain pathways due to genetic alterations (Fig. 4). Nanki et al. investigated phenotype-genotype correlations by focusing on niche factor dependencies and occurred genetic aberrations [83]. *Ki-ras2 kirsten rat sarcoma viral oncogene homolog (KRAS)* mutation and RTK amplifications like erb-b2 receptor tyrosine kinase 2 (ERBB2) or erb-b2 receptor tyrosine kinase 3 (ERBB3) led to the acquisition of EGF and FGF growth factor independency (Fig. 4A). The upregulation of epiregulin (EREG), a ligand of EGFR, mediated EGF and FGF independency suggesting an EREG autocrine loop for pathway activation (Fig. 4A) [83].

Gastric organoid growth is dependent on TGF-β and BMP signaling inhibition. However, TGF-β and BMP4 treatment of organoids carrying mutations in the *transforming growth factor beta receptor 2 (TGFBR2)* and the *smad family member 4 (SMAD4)* did not influence proliferation rate of organoids (Fig. 4B). Some gastric cancer organoids with no alterations in the aforementioned genes also tolerated stimulation with TGF-β and BMP4, hinting to additional nongenetic mechanisms that lead to the tolerance of PDOs toward TGF-β and BMP signaling [83, 113].

WNT and Rspondin are essential for successful normal gastric organoid growth (Fig. 4C, D). Therefore, gastric cancer organoids often acquire WNT pathway independency during tumorigenic progression e.g., by *adenomatous polyposis coli (APC)* gene mutations [81, 83]. Another mechanism constitutes the upregulation of WNT ligand production, leading to an autocrine self-stimulation of the tumor (Fig. 4C). This mechanism can be suppressed by treatment with the WNT ligand production inhibitor porcupine [114].

Among the WNT ligand dependent PDOs some represented an additionally unique Rspondin independency. Rspondin normally binds to LGR4/5 stabilizing the WNT receptors Frizzled and LRP (Fig. 4D). In the absence of Rspondin, RNF43 as well as ZNRF3 are ubiquitinating the WNT receptors [58, 59, 115]. *RNF43* mutations are found in only 5% of microsatellite stable tumors, but have a high frequency of 55% in MSI subtype patients [116]. Interestingly, some stomach and intestinal cancer organoids with single *RNF43* mutations were still Rspondin dependent [58, 83]. Subsequent genetic analyses revealed that double mutations, homozygous deletions as well as mRNA downregulation of *RNF43* and the corresponding homolog *ZNFR3* resulted in Rspondin independency. Interestingly, this was additionally seen for a *RNF43*^*D300Y*^ single mutation (Fig. 4D).

#### Drug response testing

**Fig. 5:**
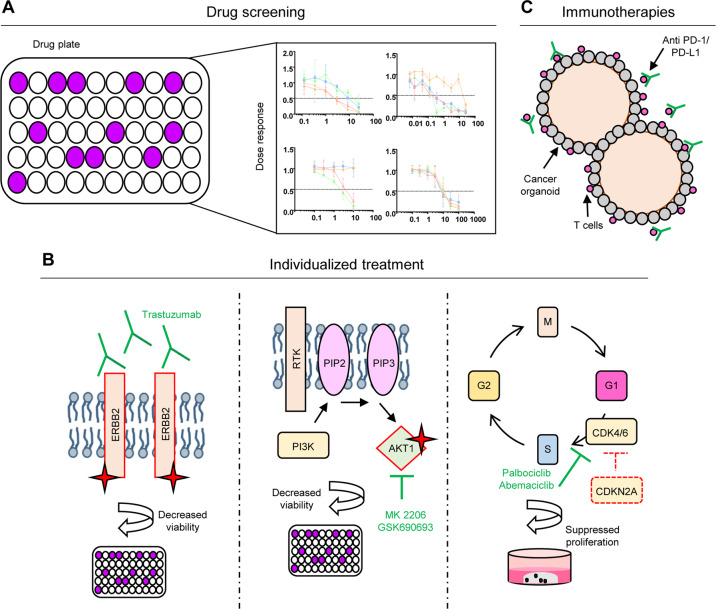
Drug response testing. **A** General drug-screening setup with dose-response curves. **B** Targeted therapy options of PDOs depending on the present mutations. ERBB2 amplifications and/or mutations can be targeted with the monoclonal antibody trastuzumab. Aberrant PI3K signaling can be inhibited using the small molecules MK-2206 and GSK690693 targeting AKT1. Loss of the tumor suppressor CDKN2A results in an uncontrolled cell cycle. Resulting proliferation can be suppressed by treatment with palbociclib and abemaciclib. **c** Immunotherapeutic treatments can be tested in co-cultures of PDOs with reactive T-cells.

Treating gastric cancer PDOs with chemotherapeutic drugs frequently used in gastric cancer treatment resulted in varying degrees of response, comparable in its spectrum to clinical responses of patients [81, 84]. In addition, PDOs allow small to medium size drug screens that might identify additional vulnerabilities (Fig. 5A) [79]. Also gastric cancer PDOs have been exposed to such screens, resulting in interesting divergent response patterns between individual PDO lines [82, 84].

Besides classical chemotherapeutics and time and cost consuming drug screens individual PDOs can also be treated with targeted drugs against identified molecular alterations in i.e., sequencing data. Amplifications of *ERBB2* are found in 22% of gastric cancer patients and patients carrying such a amplification can be successfully treated with trastuzumab [37]. Accordingly, response could be documented for a gastric cancer PDO line with an *ERBB2* amplification, as well as in an PDO with an *ERBB2* pathway activating mutation [81]. Mutations in the *AKT serine/threonine kinase 1* (*AKT1*) gene resulted in a strong response to treatment with MK-2206 and GSK690693, both inhibitors of AKT [82]. Alterations in the tumor suppressor *CDKN2A*, frequently observed in gastric cancer (34%), play a key role in tumorigenic cell cycle progression [39]. Loss of the gene results in a permanently active cell cycle and therefore aberrant proliferation. Treatment with palbociclib or abemaciclib, both *cyclin dependent kinase 4/6* (*CDK4/6*) inhibitors, resulted in a suppression of proliferation (Fig. 5B) [81, 84].

The use of organoids in personalized medicine, i.e., to delineate treatment strategies for individual patients; depends on the rate of correct prediction of response. In order to ascertain the predictive value of PDOs, co-clinical trials are needed to document side-by-side the clinical effect in vivo and the effect in organoids in vitro. The first study to document such correlations was carried out by the group of Nicola Valeri [82]. Overall, a strikingly high sensitivity, specificity as well as positive and negative predictive value or organoids to predict response to targeted drugs could be documented.

Taken together, first data indicate the usefulness of patient derived organoids in therapy prediction. In our experience, cancer organoids can be established from biopsies within 2–3 weeks for ~50% of patients to an extend that allows to test responses to standard chemotherapeutics. This is similar to the time frame of the routine work (i.e., CT and laparoscopic staging, intravenous port implantation) that happens before starting the neoadjuvant chemotherapy. Patients in a palliative setting normally receive first line of chemotherapy according to established protocols, before further personalized approaches are applied. This usually gives enough time to expand cultures to perform drug screens, i.e., in the form of a high-throughput drug screen as established by Du et al., before suggestions for second/third line treatment regimens are needed [117]. In our view, it will therefore be possible in the future to integrate PDOs into the clinical decision-making process. Of note, Yan et al. reported a heterogeneous drug response between PDOs from different tumor regions of one tumor [84]. Further co-clinical and first prospective studies in larger patient cohorts are therefore needed to clearly define the role of PDOs in different therapy settings (i.e., neoadjuvant/palliative). In addition, for novel immunotherapeutic approaches, co-culture systems with immune cells also need to be validated concerning in vitro functionality of tumor-immune cell interaction [118]. First promising data hint toward the possibility for functional testing of e.g., checkpoint inhibition therapies using immune cell-organoid co-cultures (Fig. 5C) [119].

Finally, for further interest we would like to direct the readers to other excellent reviews on different aspects of the topic of organoids [40, 100, 120–130]. The generation of tumor organoid libraries from other entities besides gastric cancer is reviewed in several articles [131–136].

## Summary and future perspectives

Organoids open up new possibilities in characterization and understanding development, tissue homeostasis, and diseases. Organoids from normal tissues are used in a wide range of laboratory applications like CRISPR/Cas9 gene editing, bacterial or viral infections as well as transplantation assays. Large human cancer organoid biobanks from different cancer entities have been established and characterized in detail. Organoid libraries provide a human cancer resource to analyze cancer biology in living cells. Individualizing cancer treatment based on drug response testing has become feasible. In the future, patient derived organoids can bridge the gap between molecular genetics, current biological understanding, and clinical therapy.
