# Correction: Gastric organoids—an in vitro model system for the study of gastric development and road to personalized medicine

**DOI:** 10.1038/s41418-021-00835-7

**Published:** 2021-08-18

**Authors:** Therese Seidlitz, Bon-Kyoung Koo, Daniel E. Stange

**Affiliations:** 1 Department of Visceral, Thoracic and Vascular Surgery, Medical Faculty and University Hospital Carl Gustav Carus, Technische Universität Dresden, Dresden, Germany; 2 Institute of Molecular Biotechnology of the Austrian Academy of Sciences, Vienna Biocenter (VBC), Vienna, Austria; 3 National Center for Tumor Diseases (NCT), Dresden, Germany: German Cancer Research Center (DKFZ), Heidelberg, Germany; Faculty of Medicine and University Hospital Carl Gustav Carus, Technische Universität Dresden, Dresden, Germany; Helmholtz-Zentrum Dresden - Rossendorf (HZDR), Dresden, Germany

**Keywords:** Cancer models, Experimental models of disease

Correction to: *Cell Death & Differentiation* 10.1038/s41418-020-00662-2, published online 22 November 2020

The article Gastric organoids—an in vitro model system for the study of gastric development and road to personalized medicine, written by Therese Seidlitz, Bon-Kyoung Koo and Daniel E. Stange, was originally published electronically on the publisher’s internet portal on 22 November 2020 without open access. With the author(s)’ decision to opt for Open Choice the copyright of the article changed on 8 July 2021 to © The Author(s) 2021 and the article is forthwith distributed under a Creative Commons Attribution 4.0 International License, which permits use, sharing, adaptation, distribution and reproduction in any medium or format, as long as you give appropriate credit to the original author(s) and the source, provide a link to the Creative Commons licence, and indicate if changes were made. The images or other third party material in this article are included in the article’s Creative Commons licence, unless indicated otherwise in a credit line to the material. If material is not included in the article’s Creative Commons licence and your intended use is not permitted by statutory regulation or exceeds the permitted use, you will need to obtain permission directly from the copyright holder. To view a copy of this licence, visit http://creativecommons.org/licenses/by/4.0/.

